# The Institutional Worker

**Published:** 1912-10-12

**Authors:** 


THE
### INSTITUTIONAL WORKER
**Being a Special Supplement to "The Hospital"**

### Editor's Notices.

**Contributions:**

Contributions should be written, or preferably typed, on one side of the paper only, and all articles sent in are accepted upon the distinct understanding that they are forwarded to THE HOSPITAL only.

The Editor cannot undertake to return MSS. not used, but when a stamped directed envelope is enclosed the MSS. may be returned if a special request is made.

Accepted articles and paragraphs of news will be paid for after publication at the scale rate.

**Address:**

To prevent delay all contributions and letters on editorial business must be addressed exclusively to the Editor, "The Hospital," 29 Southampton Street, Strand, London, W.C. It is important that this regulation shall be strictly observed.

**Correspondence:**

Correspondence on all subjects is invited. The name and address of correspondents must be given as a guarantee of good faith, but not necessarily for publication.

**Special Articles:**

Special articles are invited, and questions, inquiries, and paragraphs upon all matters relating to the work, administration, and management of general, special, mental, fever, cottage and convalescent hospitals, sanatoria, homes, institutions, societies and organisations for the treatment or care of the sick, injured and dependants of all classes. Every matter affecting the interests and welfare of the staff of all grades working in these institutions will receive special consideration.

**Administrative Medicine, Research and Items of News:**

Special terms will be paid for approved articles on subjects in this wide field by experts who have made some section of it their special study and interest. We wish to give prominence to every movement tending to advance accuracy of diagnosis, efficiency in treatment, and the development of modern methods to eradicate disease and promote the welfare of the suffering.

**A Bureau of Information:**

We invite inquiries and applications for information and help in providing for that numerous class of sufferers who require special care or house-room which they cannot obtain in their own homes or secure for themselves. **We cannot, however, prescribe, or recommend practitioners.**

**Books for Review:**

Publishers are particularly requested to send advance proofs of any new books of importance whenever possible, as the Editor has made arrangements to publish immediate reviews on a new plan.

**Photographs, Plans, Blocks, and Illustrations:**

It is requested that wherever possible MSS. may be accompanied by illustrations in any of the above forms. The name of the sender and of the article to which it belongs should be written on the back of each photograph, drawing, or block for purposes of identification.

**Local Papers:**

Newspapers containing reports or news paragraphs should be marked and addressed to the Sub-Editor.

**The Coupon System:**

A coupon will be found at the bottom of the third inside page of the cover each week. This coupon must be attached to every question or inquiry to which an answer is desired in THE HOSPITAL.

### It is to Your Interest to send for the Booklet containing full particulars of THE HOSPITAL Bureau of Information.

It is the purpose of this Department to become a practical help to *every* Institutional Worker, Health Visitor, District Nurse, and all associated with the Prevention of Disease, Care of the Sick, and the Advancement of National Health.

Years of experience in Institutional Affairs and the Medical Health and Sanitary Services are placed at the disposal of every reader of THE HOSPITAL requiring assistance entirely without charge.

The want of accurate information on the many phases of Institutional Work has been the stumbling-block in the past, but with the inauguration of THE HOSPITAL Bureau of Information this need be no longer a bar to progress.

A knowledge, therefore, of the scope and work of the Bureau is a matter that deeply concerns your Professional career, and it is to your interest to obtain at once details of this new development of THE HOSPITAL, THE Journal of the Institutional Worker.

All letters in regard to the Bureau must be addressed to THE HOSPITAL Bureau of Information, THE HOSPITAL Building, 28-29 Southampton Street, Strand, London, W.C.

### Manager's Notices.

**All letters on business matters must be addressed to The Manager, "The Hospital," 28 and 29 Southampton Street, London, W.C.**

**Advertisements:**

To ensure insertion, all advertisements for THE HOSPITAL must reach the Manager not later than Wednesday at noon in each week. For scale of charges see pages vii and 2.

**Remittances:**

**It is especially requested that remittances be made by Cheque or Postal Order, and in halfpenny stamps only when the amount is under sixpence.**

**Subscriptions,** which may commence at any time, are payable in advance. Cheques and Post-Office Orders should be crossed London County and Westminster Bank, Covent Garden Branch, and made payable to the Manager of THE HOSPITAL.

RATES OF SUBSCRIPTION (including Postage).

| *United Kingdom.* | | *Foreign and Colonial.* | |
|---|---|---|---|
| Three Months ... | ... 2s. 0d. | Three Months ... | ... 2s. 6d. |
| Six Months ... | ... 4s. 0d. | Six Months ... | ... 5s. 0d. |
| Twelve Months ... | ... 6s. 6d. | Twelve Months ... | ... 8s. 8d. |

*SUBSCRIPTION FORM.*

*Please send me* THE HOSPITAL *for*..................*months, commencing with your issue of*............................, *for which please find enclosed*............................

*Name*..........................................................

*Address* ..........................................................

..........................................................

*Date*........................................

*To* ........................................ *Newsagent.*

Or to

*The Manager,*
THE HOSPITAL,
The Hospital Building,
28/29 Southampton Street,
Strand, London, W.C.

### The Scientific Press Publications

**LEDGERS, ACCOUNT BOOKS, STOCK BOOKS of all kinds for large and small Institutions.**

These are supplied already ruled and printed, or in accordance with the special requirements of institution managers.

### TEMPERATURE CHARTS.

MORNING, EVENING, FOUR-HOUR, MATERNITY.

Size 10 inches by 8 inches 12 3d. 25 6d. 50 9d. 100 1/4 | 250 3/3 500 6/6 1,000 12/6

Postage extra.

All Orders for 250 and over are sent Carriage Paid.

### CASE PAPERS

For Day and Night Nurse's Reports. Bound & unbound.

### DISTRICT NURSING ASSOCIATIONS'

Case, Visit, Time, and Loan and Account Books.

### INSTITUTIONAL LIBRARY.

THE SCIENTIFIC PRESS will procure literature, professional and otherwise, for the formation of and addition to Institutional Libraries. It is economy of time and expenditure to utilise one source of supply.

**THE SCIENTIFIC PRESS, LTD.,**

**28 and 29 Southampton Street, Strand, London, W.C.**

SEND FOR COMPLETE CATALOGUE.

